# A benchmark study of sequence alignment methods for protein clustering

**DOI:** 10.1186/s12859-018-2524-4

**Published:** 2018-12-31

**Authors:** Yingying Wang, Hongyan Wu, Yunpeng Cai

**Affiliations:** Research Center for Biomedical Information Technology, Shenzhen Institutes of Advanced Technologies, Chinese Academy of Sciences, Shenzhen, China

**Keywords:** Multiple sequence alignment, Pair-wise sequence alignment, Benchmark

## Abstract

**Background:**

Protein sequence alignment analyses have become a crucial step for many bioinformatics studies during the past decades. Multiple sequence alignment (MSA) and pair-wise sequence alignment (PSA) are two major approaches in sequence alignment. Former benchmark studies revealed drawbacks of MSA methods on nucleotide sequence alignments. To test whether similar drawbacks also influence protein sequence alignment analyses, we propose a new benchmark framework for protein clustering based on cluster validity. This new framework directly reflects the biological ground truth of the application scenarios that adopt sequence alignments, and evaluates the alignment quality according to the achievement of the biological goal, rather than the comparison on sequence level only, which averts the biases introduced by alignment scores or manual alignment templates. Compared with former studies, we calculate the cluster validity score based on sequence distances instead of clustering results. This strategy could avoid the influence brought by different clustering methods thus make results more dependable.

**Results:**

Results showed that PSA methods performed better than MSA methods on most of the BAliBASE benchmark datasets. Analyses on the 80 re-sampled benchmark datasets constructed by randomly choosing 90% of each dataset 10 times showed similar results.

**Conclusions:**

These results validated that the drawbacks of MSA methods revealed in nucleotide level also existed in protein sequence alignment analyses and affect the accuracy of results.

**Electronic supplementary material:**

The online version of this article (10.1186/s12859-018-2524-4) contains supplementary material, which is available to authorized users.

## Background

Protein sequence alignments, as an effective and intuitive way of identifying homologous regions among sequences, play a fundamental role in various biomedical researches such as database construction and query, prediction of protein structure and function, etc. [1]. Protein sequence alignments could identify regions of similarity that may reflect biological relationships among the input sequences. As a result, protein sequence alignments analyses become a crucial step for many bioinformatics analysis studies during the past decades. Many protein databases covered protein family information had been built based on sequence alignments such as PROSITE [2], Pfam [3], and ProDom [4], etc. In the field of database query, protein sequence alignment algorithms such as BLAST [5], FASTA [6], dynamic programming methods [7] and other methods [8–10] enable researchers to compare a query protein sequence with databases or library to get similar sequences of the input sequence. Sequence alignment could detect motifs and important functional or structural residues such as binding sites, etc. [11–15]. Such information got from sequence alignment analyses could be used to map onto protein 3D structure and help deducing potential function of the protein [16, 17].

Various kinds of methods have been proposed for creating an alignment, including pair-wise sequence alignment (PSA), multiple sequence alignments (MSA), profile-based methods, prediction-based methods, and structure-based methods, etc. Of which, PSA and MSA are most widely used. PSA aligns each pair of sequences once at a time. It is the simplest form of an alignment which can be performed with two approaches: global or local. The computational definition of PSA is to find the alignment that maximizes the two input protein sequences’ similarity. PSA methods are usually used to calculate the sequence similarity on function, structure and/or evolution levels [7, 18]. Many PSA methods have been developed such as EMBOSS [19], BLAST [20], CD-HIT [21], ESPRIT [22], and UCLUST [23], etc. The procedure of MSA can also be performed with global and/or local approaches and it is more complex: the MSA methods arrange multiple protein sequences into a rectangular array aiming to make residues in a same column homologous or with similar functions. Many traditional methods have been developed such as MUSCLE [24, 25], MAFFT [26, 27], CLUSTALW [28], Clustal Omega [29], ProbCons [30], T-Coffee [31], PROMALS [32], SPEM [33], Expresso [34], PROMALS3D [35], Align-M [36], KAlign [37], DIALIGN [38], POA [39], HAlign [40], ProDA [41], 3DCOFFEE [42], NAST [43], and Mothur [44], etc. These MSA methods are often used to assess sequence conservation, tertiary and secondary structures of protein. Homology and evolutionary relationships could be inferred from the output of MSA methods because there is an underlying assumption for MSA: all the sequences to be aligned may share recognizable evolutionary homology.

The reliability of alignment results is an indispensable prerequisite for most downstream analyses. Nevertheless, it has been observed that the alignment results produced by different tools can be quite diversified [45]. Evaluation of sequence alignment methods is often quite a complicated problem due to the unavailability of ground truth. As a result, systematic benchmarks that provide clear guidance about the capabilities and limitations of these sequences alignment algorithms are crucial since the conclusions are based on objective, quantitative comparisons [46]. Early in 1994, a study compared the ability of different MSA methods of finding the highly conserved functional motifs throughout a given protein family. Due to the limitation of datasets, this study used only four protein family as test datasets [47]. Constructing standard, high-quality protein sequences’ benchmark datasets become a crucial step in the fields. Some datasets were developed to solve such problems as follows: BAliBASE datasets were constructed based on 3D structural super-positions that were manually refined to ensure the correct alignment of conserved residues [48–51]. HOMSTRAD [52] provided combined protein sequence and structure information extracted from PDB [53], Pfam [3] and SCOP [54], and so on. OXBench was built automatically using structure and sequence alignment methods and was divided into three datasets [55]. SABmark was built based on sequences derived from the SCOP protein structure classification and it only provided ‘gold standard’ alignments for pairs of sequences [56]. Prefab was built using a fully automatic protocol and pairs of sequences with known 3D structures were selected and aligned [24]. IRMBase was designed to test local multiple alignment methods and the benchmark datasets were synthesized [57, 58].

Several studies have focused on the performance of MSA method using these benchmark datasets [59–64] by analyzing the alignment accuracy [65, 66], computing time and memory usage [67], etc. The results of these studies indicated that there all MSA methods have their own strengths and weaknesses and no MSA method was perfect on all benchmark datasets. However, these indicated us that there were some common drawbacks among all the MSA methods. Uncertainty was one disadvantage of MSA methods that cannot be ignored. A study showed that uncertainty in the MSA alignments can lead to several problems, including different alignment methods resulting in different conclusions [45]. The presence of a large proportion of highly diverse sequences was shown to affect the alignment of sequences with a small genetic distance while using MSA methods [22]. Negative effects on clustering results were another kind of drawback when compared with PSA methods. A study showed that MSA-based clustering methods get worse results than PSA-based clustering methods on 16 s rRNA datasets [68].

The reasons causing these drawbacks of MSA methods may be as follows: (1) The evolutionary relationships of input sequences were often unknown thus the assumption of MSA methods were not met. (2) The design of some MSA methods’ alignment evaluation scores focused on math sense instead of biological meanings. (3) Many MSA methods adopted heuristic search in order to deal with massive sequences which made themselves easier to fall into local optimization. Compared with this, PSA methods could identify similar regions of protein sequences in a fast and flexible way when applied on nucleotide level [69, 70]. Thus it is valuable to test whether such conditions also exists on protein level by comparing MSA and PSA methods in a systematically way.

Normally, a benchmark study is based on some kind of understanding of what the correct result should be, thus a specific and significant definition of what ‘correct’ or ‘gold standard’ and measures used to reflect the results are crucial. Two commonly used scores in MSA benchmark studies are Sum-of Pairs score (SP) and Column Score (CS). SP increases with the number of correctly aligned sequences and is used to determine the extent of MSAs succeed in an alignment. CS is a binary score that shows the ability of MSA methods to align all the input sequences correctly. However, SP and CS only consider the correctly aligned residues. To overcome this limitation, an alternative approach is Position Shift Error (PSE) score, which is used to measure the average magnitude of error. This score could ensure misalignments that caused a small shift between two sequences are penalized less than large shifts. Other metrics such as fD and fM have been developed to distinguish the regions that were homologous from the unrelated regions. These metrics may reflect the ability of MSA methods through a computational perspective; however, the underlying assumption is that all the input protein sequences are globally align-able, which means that only substitutions, small insertions, and deletions are considered to be the mutational events separating those protein sequences. However, most protein benchmark datasets are grouped into different sub-datasets which contain several protein families. This indicated us that the ‘correct’ or ‘gold standard’ results should be consistent with the protein family divided on biological levels. Each benchmark dataset contain several protein families which could be considered as classes and the proteins in them can be considered as samples with known class labels. Each benchmark dataset could thus be considered as a clustering result and the ‘correct’ results given by MSA or PSA methods should be the one best fit with it. Cluster validity criteria which are quantitative measures are suitable here [71] to evaluate the fitness between results generated from MSA or PSA methods and the correct results (real protein family divisions). A higher cluster validity value means the corresponding alignment method shows better performance. Several cluster validity measures have been developed to assess the quality of clustering algorithms such as Dunn and Dunn like Indices [72], Davies Bouldin Index [73], SD Validity Index [74], S_Dbw Validity Index [75], Silhouette Width [76], and R Squared index [77]. Dunn is time consuming and very sensitive to noise since the score is closely related to the maximum and minimum distances between samples. Davies Bouldin Index, SD Validity Index, and S_Dbw Validity Index need to choose a representative point from each cluster.

In this paper we propose a new benchmark framework for protein sequence alignment methods based on cluster validity. This new framework directly reflects the biological ground truth of the application scenarios that adopt sequence alignments, and evaluates the alignment quality according to the achievement of the biological goal, rather than the comparison on sequence level only, which averts the biases introduced by alignment scores or manual alignment templates. In contrast to former studies, we calculated the cluster validity scores based on sequence distances directly instead of clustering results, which avoids the influence brought by different clustering methods, and makes the comparison fairer for both MSA and PSA methods. Results showed that PSA methods have higher cluster validity score than MSA methods on most of the benchmark datasets. These results validated that the drawbacks of MSA methods revealed in nucleotide level also existed in protein sequence alignment analyses and affect the accuracy of results.

## Methods

**Fig. 1:**
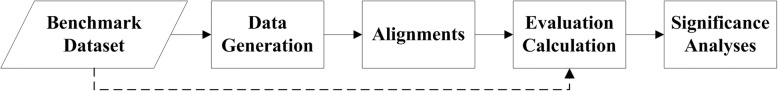
Framework of this benchmark study This benchmark study is performed following four main steps including data generation, alignments, evaluation calculation, and significance analyses

Figure 1 depicts the pipeline of the benchmark procedure carried out in this paper, which comprises four main steps as follows: (1) Data Generation; (2) Alignment Analyses; (3) Evaluation Calculation; (4) Significance Analyses. Firstly, sequences with different class labels were combined to generate each benchmark dataset. Then, the alignment analyses were performed using 6 MSA and 1 PSA methods and the aligned sequence matrices with gaps inserted are generated as outputs. Based on this, evaluation calculation was performed by cluster validity calculation using SW and RS scores, based on distances calculation results. Finally, Significance analyses were performed on biological and statistical levels to determine whether the performance differences between algorithms produces essential discriminations on application scope.

One major difficulty for comparing alignment methods against biological backgrounds is that de-novo sequence binning relies heavily on the choice of clustering methods, which is independent of the alignment itself but greatly impacts the outcome. To avert this influence, we adopt a clustering-free approach on the evaluation step. Instead of creating clusters and matching them with real taxonomy, we directly evaluate how the taxa are separated by the alignment results. An optimal alignment will be expected to maximally separate sequences of different family, while on the other hand group sequences of the same family together. In this manner the alignment quality of different algorithms are evaluated.

### Data generation

We downloaded eight datasets from BAliBase v3.0. Two steps of analyses were performed on these datasets to generate eight groups of benchmark datasets as follows: (1) For each dataset, we combined protein sequences of different protein families into one file. All the protein families and sequences were included in the analyses except for the sequences belonged to more than one protein families since they would cause the confusion of the following steps. Each protein sequence was considered as a sample and the family it belonged to was considered as the sample’s class label. The original family labels of the sequences are considered as the ground truth of the clustering results. (2) For each dataset, we randomly chose 90% of the sequences from the file generated in the above step to construct a re-sampled benchmark dataset. This procedure was repeated 10 times for each dataset. Thus eight groups of benchmark datasets were generated and each group contained 11 datasets including one benchmark datasets downloaded from BAliBase and 10 re-sampled datasets. The details of these eight benchmark datasets groups were listed as follows and Table 1.

**Table 1:** Benchmark datasets list

Reference Name	Dataset ID^a^	Number of sequences^b^	Number of classes^c^	Average length
Reference1	RV11	236	38	301.178
	RV12	382	44	392.6885
Reference2	RV20	1706	41	384.3581
Reference3	RV30	1723	30	387.9745
Reference4	RV40	1113	49	480.0952
Reference5	RV50	443	16	516.6546
Reference9	RV911	423	29	701.5792
	RV912	228	28	454.0351

^a^Dataset IDs are abbreviation for the datasets and are used to refer the corresponding dataset in this paper. ^b^Number of sequences means the number of sequences with only one class label in the raw datasets. ^c^Number of classes means the number of pre-defined protein clusters in each benchmark dataset

Reference 1 (including RV11 and RV12) contained full-length equidistant sequences with two different levels of conservation: RV11 contained very divergent sequences with < 20% residue identity and any two sequences shared 20–40% residue identity were included in the dataset RV12. Sequences having large internal insertions or extensions were excluded. The average number of sequences (309) and the average sequence length (346.9332) in this dataset were both the smallest of all the datasets

Reference 2 (RV20) contained full-length families aligned with a highly divergent ‘orphan’ sequence. A family was included in this dataset if all the sequences shared > 40% residue identity and for which at least one 3D structure was known. ‘Orphan’ was referred to a sequence that shared < 20% residue identity with all members in the family. The numbers of sequences in the dataset was 1706 with average sequence length 384.3581

Reference 3 (RV30) contained full-length sequences with < 25% residue identity between any two sequences from different families. It was designed to demonstrate the ability of the programs to correctly align equidistant divergent families into a single alignment. The numbers of sequences in the dataset was 1723 with average sequence length 387.9745

For References 1–3, the percent identity was calculated over the homologous region only, and no sequences contain large internal insertions.

References 4 (RV40) and 5 (RV50) contained full-length sequences shared > 20% residue identity that contained large N/C-terminal extensions or internal insertions respectively. The two datasets were designed to evaluate a program’s ability to identify the presence of the insertions not to judge the overall quality of an alignment. The numbers of sequences in the two datasets were 1113 and 443 with average sequence length 480.0952 and 516.6546

Reference 9 (including RV911 and RV912) contained full-length sequences with linear motif alignment. RV911 contained sequences with < 20% residue identity and RV912 contained sequences with 20–40% residue identity. The average number of sequences was 325.5 and the average sequence length was 577.8071 in this dataset

### Alignment methods tested

Six MSA programs including MUSCLE (default), MUSCLE (iters = 2), MAFFT (FFT-NS-2), MAFFT (L-INS-i), Clustal Omega, and KAlign were chosen based on different algorithmic approaches beyond download availability and popularity. ESPRIT was used on behalf of PSA methods. The details of these methods were listed as follows:

#### MSA method-MUSCLE (MUltiple Sequence Comparison by Log-Expectation)

MUSCLE had three stages: draft progressive, improved progressive, and refinement. The first stage calculated the similarity of each pair of input sequences using k-mer counting or by constructing a global alignment of the pair to get a triangular distance matrix constructed a tree based on it. After this, a progressive alignment was built. The second stage attempted to improve the tree constructed in the first step and built a new progressive alignment according to this tree. The third stage performed iterative refinement using a variant of tree-dependent restricted partitioning. At the completion of each stage, a multiple alignment was available and the algorithm can be terminated. MUSCLE was shown to be suitable for medium alignments. In this paper, we chose two parameter settings of MUSCLE based on the consideration of accuracy and speed: MUSCLE (default) and MUSCLE (iters = 2). The default settings were designed for best accuracy rather than making any compromises for speed, and the option ‘iters = 2’ (short for maxiters two Iterations) was designed for large datasets where long execution times becomes an issue.

#### MSA method-MAFFT (Multiple sequence Alignment based on the Fast Fourier Transform)

MAFFT offered various multiple alignment strategies which were classified into three types: (1) progressive method (including FFT-NS-1, FFT-NS-2), (2) iterative refinement method (including FFT-NS-i, NW-NS-i), (3) iterative refinement method using both the WSP and consistency scores (including L-INS-i, E-INS-i, G-INS-i). Strategies in type1 ran the fastest in speed and strategies in type3 was the most accurate. The same as MUSCLE, we chose two parameter settings of MAFFT based on the consideration of accuracy and speed: MAFFT (FFT-NS-2) and MAFFT (L-INS-i).

#### MSA method-Clustal Omega

Clustal Omega was the latest member of the ‘Clustal family’. Compared with previous versions, Clustal Omega offered a significant increase in scalability, allowing virtually any number of protein sequences to be aligned quickly with similar accuracy of other MSA methods.

#### MSA method-KAlign

KAlign was a global, progressive alignment method which employed an approximate string-matching algorithm to calculate sequence distances and incorporated local matches into the global alignment. It was designed to deal with large-scale sequences with quickly speed and accuracy.

#### PSA method-ESPRIT

ESPRIT performed global pair-wise sequence alignment using Needleman-Wunsch algorithm.

### Evaluation calculation

To evaluate the performance of different methods we analyzed in this study, we performed evaluation calculation using three procedures: similarity calculation, distance calculation, and cluster validation calculation. The input of this step was the aligned sequence matrices generated by each alignment method and the output was a cluster validity value. The ‘distance calculation’ was calculated based on ‘similarity calculation’ while the ‘cluster validation calculation’ was calculated based on ‘distance calculation’. The detailed way of the three procedures were as follows:

#### Similarity and distance calculation

The percent identify (ID) score was used to calculate the similarity between two sequences in the aligned matrixes generated in the step named ‘alignment analyses’ as follows:

1 $$ \mathrm{ID}=\left(\mathrm{number}\ \mathrm{of}\ \mathrm{matched}\ \mathrm{residues}\right)/\left(\mathrm{whole}\ \mathrm{length}\ \mathrm{of}\ \mathrm{aligned}\ \mathrm{sequences}\right) $$

The ID score was adopted by BLAST programs and it could reflect the percentage of identical residues in the aligned sequence pairs.

Based on the similarity ID score, the distance between two protein sequences was calculated as follows:

2 $$ \mathrm{dis}=1-\mathrm{ID} $$

For PSA method, if two sequences were exactly the same, the ID score would be the maximum value 1.0 and the distance score dis will be 0. It should be noted that, however, for MSA methods, the ID score may not be the maximum value 1.0 even when two sequences are identical, because MSA algorithms may produce different alignment results for identical sequences within one run.

#### Cluster validity calculation

Since cluster validity index were designed to evaluate the fitness degree MSA or PSA aligned results and the real protein family divisions, the index should not be too sensitive to noise such as Dunn and Dunn like indices and should not add burden to the calculation such as importing the representative point for each cluster as many index required. We chose two cluster validity indices which met the above criteria: silhouette width and RS to evaluate the performance of each MSA and PSA methods.

#### SW score

Silhouette was used to find the partitioning that best fitted the underlying data and was not easily affected by noise data. If one sequence alignment method got well-clustered results, the value will near 1.0; otherwise the value will near − 1 if it was poorly-clustered results. Higher silhouette value meant intra-distances (distances among the same class) were much smaller than inter-distances (distances among different classes) which proved the partitioning to be a good one. The Silhouette Width (SW) score for a partition was calculated as

3 $$ SW=\frac{\sum_{i=1}^nS(i)}{n} $$

where for each sequence *i*, the silhouette value was defined as

4 $$ S(i)=\frac{b(i)-a(i)}{\max \left\{a(i),b(i)\right\}} $$

where *a(i)* was the average distance between sequence *i* and other sequences in the same cluster; *b(i)* was the minimum average distance between sequence *i* and any other clusters where *i* was not a member. The cluster with this minimum distance was called ‘nearest neighboring cluster’ of *i* because it was the next best fit cluster for sequence *i*.

#### RS score

RS score was used to measure the dissimilarity of clusters. The values of RS ranged from 0 to 1. A higher RS value meant better clustering. It was calculated as:

5 $$ RS=\frac{SS_t-{SS}_w}{SS_t} $$

Of which *SS*_*t*_ referred to the total sum of squares of the whole dataset, *SS*_*w*_ referred to the sum of squares within cluster. The sum of squares was calculated as ${\sum}_{k=1}^n\left({x}_k-\overline{x}\right)$, where *x*_*k*_ stood for the distance between two sequences, $\overline{x}$ stood for the mean distances of all the sequences, and *n* stood for the number of the combination among different sequences.

It should be noted that, although PSA methods are likely to produce smaller distance for a sequence pair compared with MSA methods, the above criterion is essentially fair for both type of methods. The reason is that both the SW score and the RS score are not measured by the sole sequence distances, but by the contrasts between intra-cluster and inter-cluster distances. Although PSA achieves smaller pair-wise distances, this applies to both within-cluster and between-cluster comparisons. If the intra-cluster and intra-cluster distances are shrinks by the same degree, the SW and RS scores won’t be increased.

### Significance analyses

For each benchmark group, the cluster validity results of different alignment methods calculated on the 10 re-sampled datasets were compared using *t* test. A higher *p-value* meant the performance of the two alignment methods was of no difference while a smaller *p-value* meant there were significant differences between the two alignment methods.

## Results

We performed protein sequence alignments using 6 MSA methods and 1 PSA method on the benchmark datasets. Six hundred sixteen alignment analyses were performed in total. Results showed that (1) Esprit got the highest scores on all the datasets based on SW calculation; (2) both Esprit and MUSCLE (default) got high scores based on RS calculation, Esprit performed a little better than MUSCLE (default) in total. Meanwhile, the computational time taken by Esprit was less than MUSCLE (default) as shown in Additional file 1: Table S1.

**Fig. 2:**
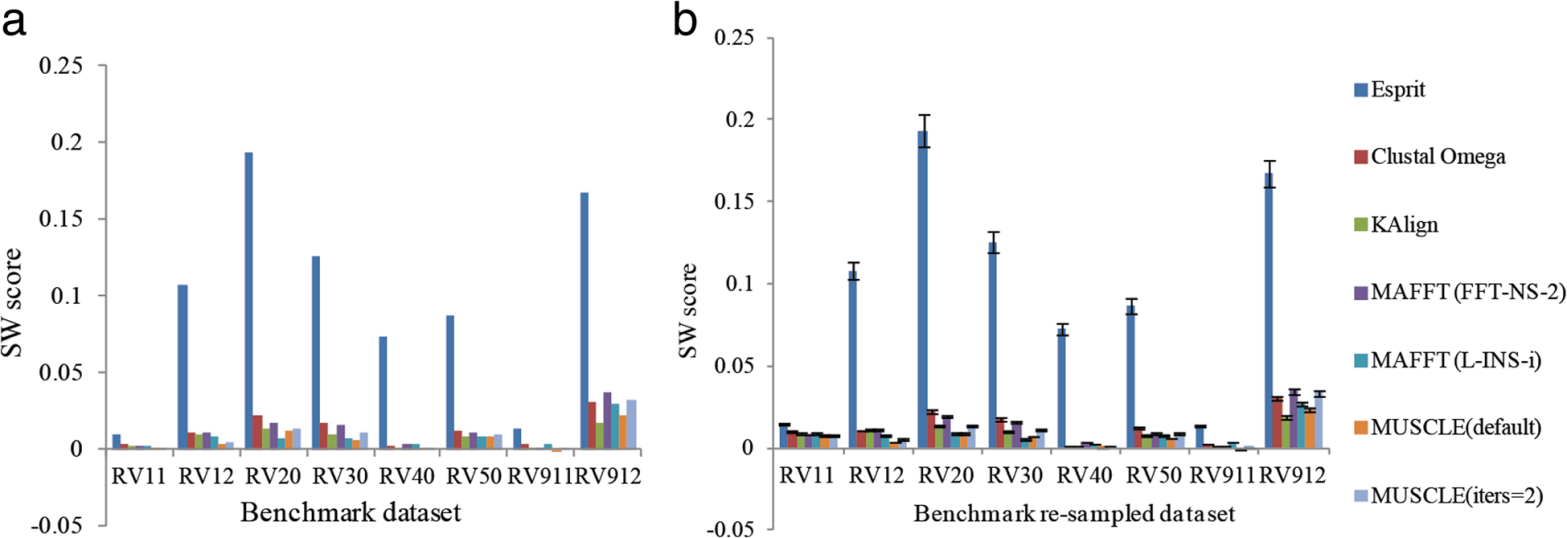
Cluster validation results based on SW score. **a** The SW score of benchmark dataset. **b** The SW scores of benchmark re-sampled benchmark dataset

Esprit got the highest SW scores on all the benchmark datasets (See Fig. 2 for details) indicating that PSA methods may be a good choice when performing protein sequence analyses. Statistical analyses showed that all the differences between Esprit and other MSA methods were significant (with small *p* values). Taken MUSCLE (default) as a representation of MSA methods, all the *p* values were less than 0.1 indicating the significant differences between these alignment methods (See Table 2 for details). The detailed results of each benchmark group were as follows:

**Table 2:** Average SW and RS scores of Esprit and MUSCLE (default) in re-sampled benchmark datasets

Re-sampled benchmark dataset	Average SW score of Esprit	Average SW score of MUSCLE (default)	*P*-value of SW	Average RS score of Esprit	Average RS score of MUSCLE (default)	*P*-value of RS
RV11	0.014383	0.007808	0.008627	0.91387	0.933349	0.5101
RV12	0.108044	0.003982	< 2.2e-16	0.705558	0.707488	0.8436
RV20	0.193411	0.009224	< 2.2e-16	0.403657	0.365046	0.07816
RV30	0.125547	0.007049	< 2.2e-16	0.407991	0.357121	0.001885
RV40	0.072819	0.000606	< 2.2e-16	0.705149	0.708882	0.7688
RV50	0.086507	0.006049	< 2.2e-16	0.322656	0.331129	0.5612
RV911	0.01329	−0.00131	3.512e-12	0.839489	0.826965	0.1271
RV912	0.167038	0.02381	< 2.2e-16	0.487026	0.37473	4.733e-07

Benchmark dataset reference1 was composed of two sub-datasets (named RV11 and RV12 in this study) which were used to reflect the abilities of alignment methods on dealing with short length sequences. Results showed that based on SW scores, Esprit performed better than other MSA methods used in this study in both RV11 and RV12 with SW scores 0.008933 and 0.107577, separately (See Fig. 2(a) for details). This indicated that the PSA methods may perform better than MSA methods when facing with short sequences. The average SW scores on the re-sampled benchmark datasets showed similar results: Esprit got the highest SW scores compared with other alignment methods in RV11 with 0.014383 and 0.108044 in RV12 (See Table 2 for details). Both RV 20 and RV30 datasets contained over a thousand of sequences which made the alignment procedures time-consuming. Esprit was the best alignment method in the two datasets with SW scores 0.193477 and 0.125665 (See Fig. 2(a) for details). The average SW scores on the re-sampled benchmark datasets showed similar results: Esprit got the highest SW scores compared with other alignment methods in RV20 with 0.193411 and 0.125547 in RV30 (See Table 2 for details). RV40 contained sequences sharing at least 20% residue identity with large N/C-terminal extensions. Esprit got the highest SW score 0.072995 in RV40 (See Fig. 2(a) for details) and the highest average SW score 0.072819 compared with other alignment methods (See Fig. 2(b) and Table 2 for details). RV50 contains sequences sharing at least 20% residue identity with internal insertions. Similar with the results of RV40, Esprit got the highest SW score 0.086898 in RV50 (See Fig. 2(a) for details) and the highest average SW score 0.086507 compared with other alignment methods (See Fig. 2(b) and Table 2 for details). RV911 is similar to RV11 for they both contain sequences sharing at most 20% residue identity. The difference is sequences in RV911 cover linear motif alignment. All the alignment methods got small SW scores and MUSCLE (default) even got a negative score (− 0.001568). The highest SW score is achieved by Esprit with 0.013568. For RV912, Esprit got the highest SW score 0.167747 (See Fig. 2(a) for details). The highest average SW scores on re-sampled benchmark datasets of RV911 and RV912 were both achieved by Esprit with value 0.01329 and 0.167038, respectively (See Fig. 2(b) and Table 2 for details).

**Fig. 3:**
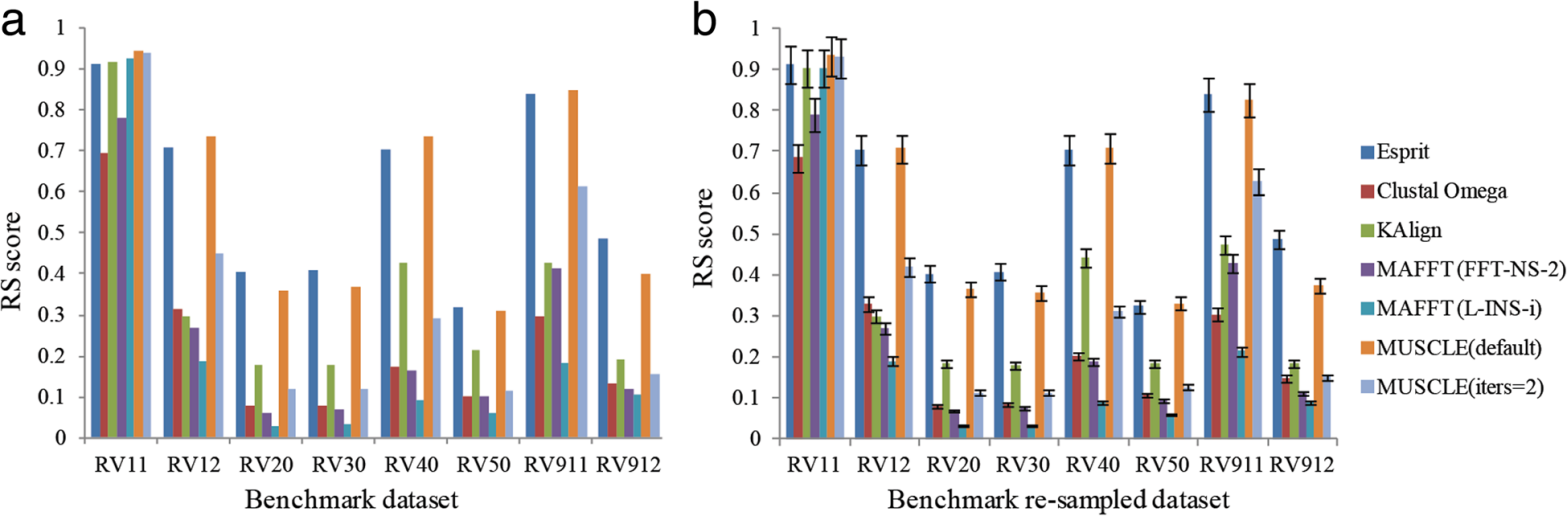
Cluster validation results based on RS score. **a** The RS score of benchmark dataset. **b** The RS scores of benchmark re-sampled benchmark dataset

Results based on RS scores showed similar results with those calculated using SW score. Since MUSCLE (default) outperformed other MSA methods by the RS scores criteria, we chose it as the representation of MSA methods as the way we analyzed the results based on SW scores. The average RS scores of Esprit and MUSCLE (default) in re-sampled benchmark datasets were listed in Table 2. The *p* values standing for the differences between Esprit and MUSCLE (default) were small (all less than 0.1) on benchmark groups RV20, RV30, and RV912 (See Table 2 for details). Esprit got the highest RS scores in these four re-sampled benchmark datasets. Compared with this, there was no significant difference between Esprit and MUSCLE (default) for re-sampled benchmark datasets RV11, RV12, RV40, RV50, and RV911 because the *p* values were at least 0.1271 (See Table 2 for details). Although the highest RS scores were achieved either by Esprit or MUSCLE (default), the results were not significant on statistical levels. This ensured that Esprit performed the best compared with other methods no matter calculated using SW or RS scores. The detailed results of each benchmark group were as follows:

The results based on RS scores showed that the performances of all methods were similar on RV11 which was the same with former researches that the resulting alignments were poor no matter which alignment method was used when dealing with diverse set of sequences. There was no statistical difference between MUSCLE (default) and Esprit on the re-sampled datasets of RV11 group, thus both of them could be considered as best performance alignment methods in this dataset group. In RV12, the highest RS score was achieved by MUSCLE (default) with 0.735538, the second highest RS score 0.708394 was achieved by Esprit. Considering the *p* value for the re-sampled datasets of this benchmark group between the two methods was not significant (with *p* value 0.8436), both MUSCLE (default) and Esprit could be considered as the best performance methods on RV12 benchmark dataset group. Same as using SW score, Esprit was the best alignment method in RV20 and RV30 (See Fig. 3(a) for details) and the results of statistical analyses also showed significant difference between Esprit and other MSA methods. These indicated that PSA methods may have better performance when dealing with family containing highly similar sequences and could align equidistant divergent families into a single alignment compared with MSA methods. MUSCLE (default) (RS score: 0.73) performed better than Esprit (RS score: 0.70) based on RS score (See Fig. 3(b) for details) on RV40. However, this result was not significant on statistical analyses since the *p* value was 0.7688 indicating both the top 2 alignment methods (MUSCLE (default) and Esprit) were good choices on RV40 benchmark dataset group. For RV50, Esprit was the best performance method with RS score 0.318475 (See Fig. 3(b) for details) compared with other alignment methods included in this study. However, the big *p* value (0.5612) indicated that both of the top 2 alignment methods (Esprit and MUSCLE (default)) were good choices when dealing with such datasets. For reference dataset 9, MUSCLE (default) got the highest RS score 0.848139 and Esprit got the second highest score (0.840775) in RV911. Similar as the results of RV40 and RV50, the big *p* value (0.1271) also indicating the best performances of both MUSCLE (default) and Esprit on RV911 benchmark dataset group. For RV912, Esprit got the highest RS score 0.485991 and the statistical analyses results was also significant (See Fig. 3(a) and Table 2 for details).

## Discussion

Considering the above results, cluster validity calculation using SW and RS scores on the datasets indicated that PSA methods perform better than MSA methods under most biological conditions. Interestingly, both Esprit and MUSCLE (default) could be considered as the best methods based on RS scores under some conditions. Due to the characters of the two scores, a higher RS score reflected the big dissimilarity among different protein families without considering the topological relationship between families; on the other hand, a higher SW score reflected a small dissimilarity inside a protein family and a clear discrimination between the family and its siblings. Hence, SW score is more sensitive and rational compared with RS score due to its definition. However, the representation of PSA methods Esprit still performed equally or better than the MSA methods analyzed in this study. These indicated that PSA methods may be a better choice if researchers focus on a balance between similarity in a same protein family and dissimilarity among different protein families which is often the option of most researchers.

## Conclusions

Protein sequence alignments are essential in many bioinformatics fields including computational analysis of protein sequences, structure modeling, functional site prediction, and sequence database searching, etc. MSA methods try to minimize the sum of pair-wise scores by aligning unrelated sequences thus the biological closely related sequences are given large distances. Compared with this, PSA could identify similar regions of protein sequences in a fast and flexible way when applied on nucleotide level.

To test the performance of MSA and PSA methods on protein sequence level, we presented a benchmark study of sequence alignment methods for protein clustering. Results showed that PSA methods performed much better than MSA methods on all the BAliBASE datasets. These indicated us that PSA methods were a better choice of dealing with protein sequence alignment analyses than MSA methods.

## Additional file

Additional file 1: **Table S1.** Relative computational time of MSAs compared to ESPRIT. (DOCX 13 kb)

## Abbreviations

CS: Column Score
ID: Percent IDentify
MSA: Multiple Sequence Alignment
PSA: Pair-wise Sequence Alignment
PSE: Position Shift Error
SP: Sum-of Pairs
SW: Silhouette Width
