# Supplementary material for: A benchmark study of sequence alignment methods for protein clustering
Source: BMC Bioinformatics. 2018 Dec 31;19(Suppl 19):529. doi: 10.1186/s12859-018-2524-4 (PMC6311937; doi:10.1186/s12859-018-2524-4)
Supplement: Supplementary file 1 — Table S1. Relative computational time of MSAs compared to ESPRIT. (DOCX 13 kb) [file 12859_2018_2524_MOESM1_ESM.docx]

|  | MUSCLE (default) | MUSCLE (iters=2) | MAFFT (FFT-NS-2) | MAFFT (L-INS-i) | Clustal Omega | KAlign | ESPRIT |
| --- | --- | --- | --- | --- | --- | --- | --- |
| RV11 | 138.73% | 8.53% | 3.41% | 580.72% | 171.92% | 3.08% | 100.00% |
| RV12 | 211.50% | 10.45% | 2.27% | 333.66% | 74.87% | 2.50% | 100.00% |
| RV20 | 172.17% | 1.80% | 0.60% | 891.91% | 38.02% | 1.24% | 100.00% |
| RV30 | 211.82% | 1.57% | 0.47% | 624.48% | 37.33% | 1.18% | 100.00% |
| RV40 | 408.69% | 8.20% | 1.68% | 1059.48% | 258.64% | 2.21% | 100.00% |
| RV50 | 116.32% | 6.18% | 1.53% | 415.83% | 94.91% | 1.75% | 100.00% |
| RV911 | 104.42% | 6.53% | 2.54% | 470.58% | 86.54% | 3.38% | 100.00% |
| RV912 | 101.79% | 7.18% | 2.37% | 276.93% | 57.55% | 3.05% | 100.00% |

Table 1 Computation time for the MSA and PSA programs
